# A prospective safety and feasibility study of metered cryospray for patients with chronic bronchitis in COPD

**DOI:** 10.1183/13993003.00556-2020

**Published:** 2020-12-17

**Authors:** Justin L. Garner, Tawimas Shaipanich, Jorine E. Hartman, Christopher M. Orton, Cielito Caneja, Karin Klooster, John Thornton, Don D. Sin, Dirk-Jan Slebos, Pallav L. Shah

**Affiliations:** 1 Department of Respiratory Medicine, Royal Brompton Hospital, London, UK; 2 Airways Diseases Section, National Heart and Lung Institute, Imperial College, London, UK; 3 Chelsea and Westminster Hospital, London, UK; 4 St Paul's Hospital, Vancouver, BC, Canada; 5 Department of Pulmonary Diseases, University of Groningen, University Medical Center Groningen, Groningen, The Netherlands

## Abstract

**Background:**

No currently approved intervention counteracts airway metaplasia and mucus hypersecretion of chronic bronchitis in COPD. However, metered cryospray (MCS) delivering liquid nitrogen to the tracheobronchial airways ablates abnormal epithelium and facilitates healthy mucosal regeneration. The objective of this study was to evaluate the feasibility, efficacy and safety of MCS in chronic bronchitis.

**Methods:**

Patients with a forced expiratory volume in 1 s of 30–80% predicted who were taking optimal medication were recruited. Primary outcomes were feasibility (completion of treatments), efficacy (3-month change in St George's Respiratory Questionnaire (SGRQ)) and safety (incidence of adverse events). Secondary outcomes were lung function, exercise capacity and additional patient-reported outcomes.

**Results:**

35 patients, 19 male/16 female, aged 47–76 years, Global Initiative for Chronic Obstructive Lung Disease grade I (n=3), II (n=10) and III (n=22), underwent staggered liquid nitrogen treatments to the tracheobronchial tree. 34 patients completed three treatments, each lasting 34.3±12.1 min, separated by 4–6 weeks; one withdrew after the first treatment. ∼1800 doses of MCS were delivered. Clinically meaningful improvements in patient-reported outcomes were observed at 3 months: change in SGRQ −6.4 (95% CI −11.4 to −1.3; p=0.01), COPD Assessment Test (CAT) −3.8 (95% CI −6.4 to −1.3; p<0.01) and Leicester Cough Questionnaire (LCQ) 21.6 (95% CI 7.3 to 35.9; p<0.01). Changes in CAT were durable to 6 months (−3.4, 95% CI −5.9 to −0.9; p=0.01); changes in SGRQ and LCQ were durable to 9 months (−6.9, 95% CI −13.0 to −0.9; p=0.03 and 13.4, 95% CI 2.1 to 24.6; p=0.02, respectively. At 12 months, 14 serious adverse events were recorded in 11 (31.4%) subjects; six (43%) moderate and eight (57%) severe. Nine were respiratory-related: six exacerbations of COPD, two pneumonias and one case of increased coughing; all recovered without sequelae. None were serious device- or procedure-related adverse events.

**Conclusion:**

MCS is safe, feasible and associated with clinically meaningful improvements in multidimensional patient-reported outcomes.

## Abstract 2

**RejuvenAir system treatment for individuals with chronic bronchitis in COPD is safe, feasible, well tolerated, and resulted in clinically meaningful improvements in multidimensional measures of cough, sputum production, breathlessness and quality of life** https://bit.ly/30KBfPs

## Introduction

COPD is a complex inflammatory lung condition characterised by airflow limitation, cough, dyspnoea and impaired quality of life [1]. Chronic bronchitis, defined as chronic cough and sputum production occurring on most days for ≥3 months of two consecutive years [2], is a common clinical phenotype of COPD [3], and is associated with accelerated lung function decline [4–6], worse health-related quality of life [7–9], increased rate of exacerbations [7, 10, 11] and hospitalisations [5, 10] and reduced life expectancy [6, 12–14].

There is no currently approved therapy that reverses the airway metaplasia and mucus hypersecretion of chronic bronchitis in COPD and restores the integrity and functionality of the respiratory tract epithelium. However, a novel approach is suggested by the observation that selective cellular ablation preserving extracellular structures is followed by rapid replacement with healthy tissue [15, 16]. Flash-freezing at −196°C induces intracellular ice crystal formation, disrupting cellular structures, but sparing the extracellular matrix, facilitating epithelial regrowth [17]. The RejuvenAir system (CSA Medical, Lexington, MA, USA) consists of a console which stores liquid nitrogen, and a disposable catheter with a radial spray head inserted through the working channel of a flexible bronchoscope. Using a specially developed algorithm, programmed doses of liquid nitrogen are delivered in a radial spray, termed metered cryospray, to the bronchial airways. It is designed to cryoablate abnormal epithelium and excessive mucous-producing goblet cells to a depth of 0.1–0.5 mm and a width up to 10 mm [18]. Re-epithelialisation with healthy mucosa has been demonstrated within 48 h of cryospray treatment, and with durability to 106 days [19].

The objective of this study was to evaluate the feasibility, efficacy and safety of MCS therapy to treat patients with chronic bronchitis in COPD. This report documents the outcomes at 12 months after the last delivered MCS treatment.

## Methods

This is a prospective, open-label, single-arm study of sequentially accrued subjects diagnosed with chronic bronchitis in COPD. The multicentre study was conducted in the United Kingdom, the Netherlands and Canada, and was approved by the respective competent authorities, institutional review boards or ethics committees at each site; all participating subjects provided written informed consent. The trial is registered at clinicaltrials.gov (NCT02483637). We recruited patients aged 47–76 years with an established diagnosis of chronic bronchitis in COPD (defined as chronic cough and sputum production occurring on most days for ≥3 months of two consecutive years) who had ceased smoking for ≥2 months prior to enrolment and had not experienced a respiratory exacerbation in the past 6 weeks, but were persistently symptomatic despite guideline-approved therapy. The extensive inclusion and exclusion criteria were designed to maximise patient safety (supplementary table S1).

### Phases of study

**FIGURE 1:**
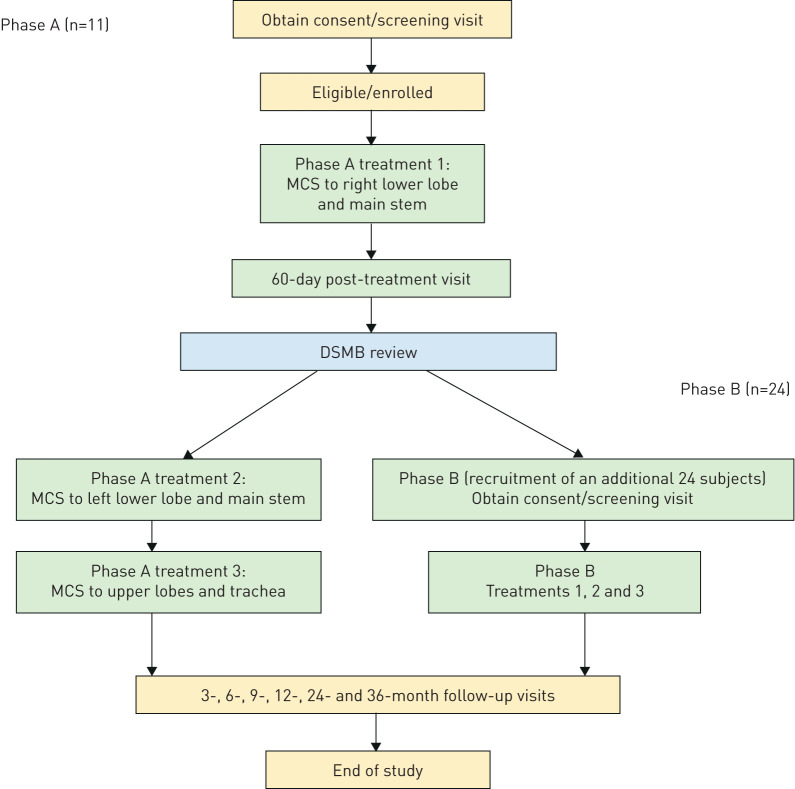
Study protocol flowchart. Each treatment is separated by 30–45 days. MCS: metered cryospray; DSMB: data safety monitoring board.

Treatments were conducted in 2 phases, which are detailed in the supplementary material (section 2.1). Phase A was a preliminary assessment of feasibility and safety and confirmation of healing, including (in this phase only) endobronchial biopsies in a small contingent of subjects undergoing their first (of three) treatments, before completing the programme of whole-lung treatments in phase B (figure 1).

Between March and August 2016, 11 subjects completed phase A. Following receipt of a satisfactory report on the findings by the data safety monitoring board (DSMB), an additional 24 subjects were enrolled and underwent the three scheduled MCS treatments; 12-month follow-up was completed in February 2019.

### Study procedures

#### Baseline and follow-up assessments

Demographics, medical history including cough and sputum production, smoking history, urine pregnancy test for females of childbearing potential, lung function (spirometry and body plethysmography), high-resolution computed tomography, 6-min walk test, plasma fibrinogen and patient-reported outcomes (including St George's Respiratory Questionnaire (SGRQ), Leicester Cough Questionnaire (LCQ); COPD Assessment Test (CAT); visual analogue score (VAS); and modified Medical Research Council (mMRC) dyspnoea scale; described in the supplementary material, section 2.2) were recorded. Subjects satisfying all the inclusion criteria proceeded to treatment. Follow-up evaluations were conducted in person at 3, 6, 9 and 12 months after completion of the final MCS treatment.

#### Device and procedure

The RejuvenAir system is a cryosurgical device that delivers metered doses of medical-grade liquid nitrogen from a Dewar stored in a console to a catheter emitting a radial spray at its tip. Details of the device and procedure have been published [17] and are outlined in the supplementary material (sections 2.3 and 2.4). General anaesthesia, sedative and associated medications were administered as per institutional guidelines and routine clinical practice.

The first treatment delivered MCS to the right lower lobe and main stem bronchus, the second to the left lower lobe and main stem bronchus, and the third to both upper lobes, any residual main stem bronchus and the distal end of the trachea. Precautionary measures were employed to avoid barotrauma and asphyxia: before each spray the cuff of the endotracheal tube was deflated, and the ventilator disconnected briefly. Chest radiography was performed 1 h post-procedure to exclude pneumothorax. The right middle lobe was omitted from the procedure on account of the perceived increased risk of barotrauma in a small structure. Endobronchial biopsies were obtained from the right lower lobe in the initial 11 patients at baseline and at day 60.

Intervals of 30–45 days were imposed between each of the three MCS treatment sessions, and progression to the next treatment was contingent on the subject remaining stable without evidence of a recent acute exacerbation.

### Study outcome measures

#### Primary outcomes

The primary feasibility end-point was the completion of all three MCS treatments. The primary safety end-point was the incidence, seriousness and relatedness of adverse events experienced during the study. The primary efficacy end-point was the change from baseline to 3 months in the SGRQ total score.

#### Secondary outcomes

Secondary end-points included changes in forced expiratory volume in 1 s (FEV_1_), 6-min walk distance and additional patient-reported outcomes (CAT, LCQ, VAS and mMRC scores).

### Statistical analyses

The sample size of 35 subjects was based on an 80% statistical power using a one-sided test at the 0.05 significance level assuming a mean±sd change of −4±7 points in total SGRQ score at 3 months relative to baseline.

Categorical data are presented as a percentage. Continuous data are summarised as mean±sd, 95% confidence interval or median (interquartile range) depending on the distribution of the data. A two-tailed paired t-test or a Wilcoxon matched-pairs signed-rank test, respectively, was used to compare these groups.

To evaluate and control for the potential effects of covariate factors on treatment outcomes, the change in SGRQ total score from baseline to 3 months was assessed using the method of least squares from an ANCOVA model incorporating baseline Global Initiative for Chronic Obstructive Lung Disease (GOLD) stage, number of MCS treatments across the three treatments (*i.e.* <50 cryosprays *versus* >50 cryosprays) and study phase.

Statistical significance was set at p<0.05 and analysis was performed using SPSS (version 24.0; IBM, Chicago, IL, USA).

## Results

Results are presented for each follow-up visit to 12 months after the completion of the last MCS treatment.

### Demographics

49 COPD subjects were screened and 35 (16 females and 19 males) enrolled in the study. The mean age was 67.2±7.0 years and BMI 26.9±5.2 kg·cm^−2^. GOLD grades were I (8.5%), II (28.5%) and III (63%). The mean duration of smoking was 56.4±35.1 pack-years (table 1).

**TABLE 1:** Baseline characteristics of patients

**Demographics**		
Age years	35	67.2±7.0
Male %	19	54.3
BMI kg·m^−2^	35	26.9±5.2
Smoking pack-years	35	45 (33 to 68)
Comorbidities	35	2 (1 to 4)
GOLD grade %		
I	3	8.5
II	10	28.5
III	22	63.0
**Baseline medications %**		
β-agonist	18	51.4
Anticholinergic	18	51.4
Corticosteroid	17	48.6
Mucolytic	6	17.1
Antibiotic	11	31.4
**Lung function**		
FEV_1_ L	35	1.4±0.5
FEV_1_ % predicted	35	50.2±14.5
FVC L	35	3.6±1.0
FVC % predicted	35	103.6±16.9
FEV_1_/FVC % predicted	35	38.5±10.1
FIV_1_ L	25	3.2±0.9
*R*_aw_ kPa·s·L^−1^	27	0.6±0.3
**Exercise capacity**		
6MWD m	35	400.6±86.8
**Symptoms**		
mMRC	35	2 (2 to 3)
CAT^#^	34	22.7±7.1
SGRQ		
Total	35	59.2±18.9
Symptoms		66.5±20.5
Impacts		48.3±22.4
Activity		74.1±19.0
LCQ	23	85.0±27.7
VAS^#^		
Rest	34	36.1±28.7
Activity	34	68.6±23.9
**Mortality score**		
BODE index	35	3 (2 to 4)
**Inflammatory marker**		
Plasma fibrinogen mg·dL^−1^	35	341.1±72.5

Data are presented as n, mean±sd or median (interquartile range), unless otherwise stated. BMI: body mass index; GOLD: Global Initiative for Chronic Obstructive Lung Disease; FEV_1_: forced expiratory volume in 1 s; FVC: forced vital capacity; FIV_1_: forced inspiratory volume in 1 s; *R*_aw_: airway resistance; 6MWD: 6-min walk distance; mMRC: modified Medical Research Council dyspnoea scale; CAT: COPD Assessment Test; SGRQ: St George's Respiratory Questionnaire; LCQ: Leicester Cough Questionnaire; VAS: visual analogue scale; BODE: BMI, airflow obstruction, dyspnoea and exercise capacity. ^#^: pre-treatment 1 data used.

At baseline, all subjects were taking at least one pulmonary medication. The most frequently used were inhaled β_2_-agonists (51.4%), anticholinergics (51.4%) and corticosteroids (48.6%). Fewer patients were taking prophylactic antibiotic (31.4%) and mucolytic (17.1%) agents (table 1).

34 patients (97.1%) attended the 3-month follow-up; 30 (85.7%) attended the 6- and 9-month follow-ups; and 31 (88.6%) were evaluated at the 12-month visit: three (8.6%) withdrew consent and one (2.9%) subject died from unrelated complications of ischaemic heart disease during this period.

### Primary outcomes

#### Primary feasibility analysis

All subjects received general anaesthesia during the bronchoscopy procedure. The mean oxyhaemoglobin saturation on room air was 98.4±1.0% at the start of treatment and 97.1±1.9% at the end of treatment.

The mean±sd number of sprays delivered was 17.3±4.6, 17.6±2.1 and 26.2±5.8 for MCS treatments 1, 2 and 3, respectively; 20.3±6.0 for all treatments. The percentage of full-dose sprays was 87.7%, 85.3% and 84.3% for treatments 1, 2 and 3, respectively; 85.8% for all treatments. The mean±sd duration of each treatment session was 34.3±12.1 min (supplementary table S2). Device observations (*i.e.* console readouts indicating the cause of spray delivery failure) were recorded in 29 subjects; the majority (95%), were related to the catheter and 5% to the console. Catheters were replaced as necessary. None of the device observations were associated with any adverse events.

All subjects were fit for discharge on the same day. Two had pre-planned stays for unrelated events. Chest radiographs were performed in all but one (2.9%) subject after treatment 1. There were no reports of pneumothoraces following any of the MCS treatment procedures.

34 (97.1%) subjects completed all three MCS treatments; one withdrew consent after experiencing a mild COPD exacerbation following the initial MCS procedure.

#### Primary safety analysis

All subjects experienced at least one adverse event (supplementary table S3). A total of 251 were reported from enrolment to the completion of the 12-month follow-up evaluation (supplementary table S4). The majority (52.6%) were classified as respiratory-related. Of these, 91 (36.3%) were attributed to the underlying COPD (supplementary table S5).

Six non-serious device-related adverse events (2.4%) were reported in four (11.4%) subjects: one episode of bronchospasm during treatment and five exacerbations of COPD occurring 1.0 (0–3.5) day after treatment and lasting 15.0 (10.5–31.0) days. These events were graded mild (n=2) or moderate (n=4) and all resolved without sequelae (supplementary table S6). There were 40 adverse events attributed to the procedure in 21 (60%) subjects; none were serious (supplementary tables S3 and S4).

14 (5.6%) serious adverse events were reported in 11 (31.4%) subjects; six (43%) were moderately serious, eight (57%) were severe (supplementary material, section 2.5). Nine were respiratory-related: six exacerbations of COPD, two pneumonias and one increased coughing. The other incidents recorded were gastritis/a duodenal ulcer, urosepsis and in one subject pulmonary embolus, rectal bleeding and ischaemic heart disease 243 days after completing all three MCS treatments. This subject was a 77-year-old Caucasian female with GOLD grade II COPD, who underwent a coronary revascularisation complicated by pancreatitis, cardiac arrest and multiple organ failure which proved fatal.

None of the serious adverse events were deemed to be related to the device or the procedure by the principal investigator or the DSMB (supplementary table S7).

The exacerbation rate from treatment 1 to 12 months was 1.84 per patient-year. Stratification according to GOLD grades II and III demonstrated rates of 1.29 and 2.10, respectively (supplementary table S8). Higher baseline SGRQ total scores were significantly associated with higher exacerbation frequency (p=0.02).

There were no reports of unanticipated adverse device effects or pneumothoraces during the study.

#### Primary efficacy analysis

**FIGURE 2:**
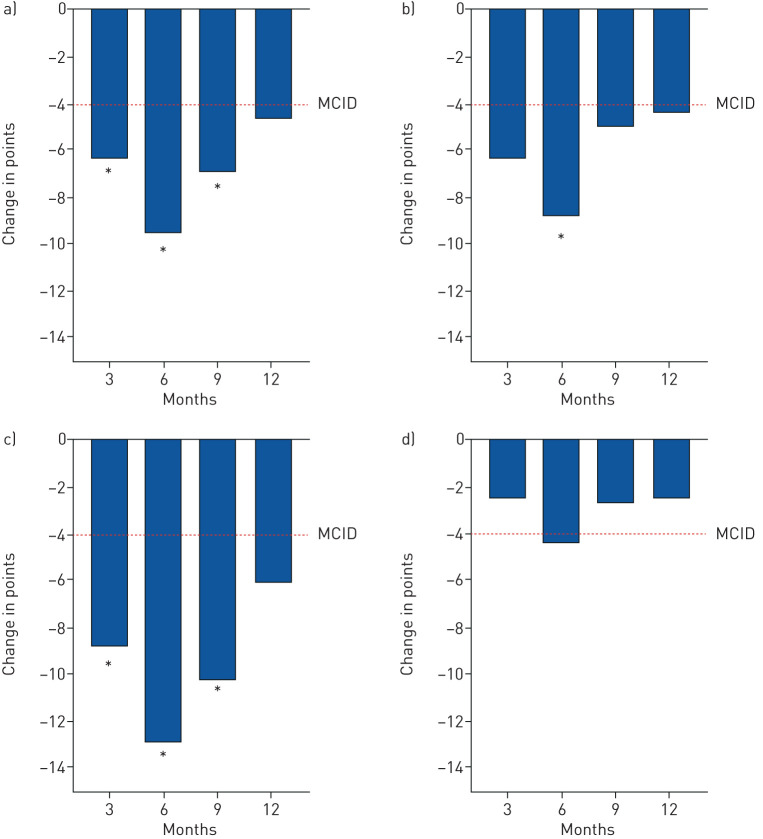
Mean changes in patient-reported outcomes over 12 months. a) St George's Respiratory Questionnaire (SGRQ) total score; b) SGRQ symptoms score; c) SGRQ impacts score; d) SGRQ activity score. MCID: minimal clinically important difference. *: p<0.05 compared to baseline.

The primary end-point, the mean change in total SGRQ score (ΔSGRQ total) from baseline to 3 months was statistically significant and clinically meaningful (≥4 points) at −6.4 (95% CI −11.4 to −1.3; p=0.01) [20], and unaffected by covariables including baseline GOLD grade, number of cryosprays administered and study phase (ANCOVA p<0.05) (figure 2 and table 2).

**TABLE 2:** Changes in clinical characteristics over 12 months

	**3 months**	**p-value**	**6** **months**	**p-value**	**9 months**	**p-value**	**12 months**	**p-value**
**Lung function**								
ΔFEV_1_ mL	−33.2±166.9 (−91.5 to 25.0)	0.25					−96.5±197.7 (−169.0 to −23.9)	**0.01**
ΔFEV_1_ %	−0.7±5.7 (−2.7 to 1.3)	0.45					−2.4±6.5 (−4.8 to 0.0)	0.05
ΔFVC mL	−125.9±330.4 (−241.2 to −10.6)	**0.03**					−191.3±483.7 (−368.7 to −13.9)	**0.04**
ΔFVC %	−3.1±9.5 (−6.4 to 0.2)	0.06					−2.8±13.0 (−7.6 to 2.0)	0.24
ΔFEV_1_/FVC %	0.3±10.6 (−3.5 to 4.0)	0.89					−0.9±3.6 (−2.2 to 0.4)	0.18
ΔFIV_1_ mL	−175.8±389.5 (−340.3 to −11.4)	**0.04**					−66.2±371.1 (−235.1 to 102.7)	0.42
ΔVC L	1.2±6.6 (−1.4 to 3.9)	0.35					−0.1±0.4 (−0.3 to 0.1)	0.49
Δ*R*_aw_ kPa·s·L^−1^	0.1±0.3 (−0.1 to 0.2)	0.28					0.0±0.2 (−0.1 to 0.2)	0.33
**Exercise capacity**								
Δ6MWD m	1.1±55.4 (−18.6 to 20.7)	0.91	20.3±72.0 (−6.6 to 47.2)	0.13	24.3±65.0 (−0.4 to 49.0)	0.05	8.5±76.2 (−19.4 to 36.5)	0.54
**Symptoms**								
ΔmMRC	0 (IQR −1 to 0)	0.29^¶^	0 (IQR −1 to 0)	0.10^¶^	0 (IQR −1 to 0)	0.16^¶^	0 (IQR −1 to 0)	0.30^¶^
ΔCAT^#^	−3.8±7.1 (−6.4 to −1.3)	**<0.01**	−3.4±6.8 (−5.9 to −0.9)	**0.01**	−0.9±7.7 (−3.8 to 2.0)	0.53	−2.0±7.2 (−4.7 to 0.6)	0.12
ΔSGRQ total score	−6.4±14.4 (−11.4 to −1.3)	**0.01**	−9.5±15.7 (−15.4 to −3.6)	**<0.01**	−6.9±16.2 (−13.0 to −0.9)	**0.03**	−4.6±15.1 (−10.2 to 0.9)	0.10
ΔSGRQ symptoms	−6.3±22.1 (−14.0 to 1.4)	0.10	−8.8±19.6 (−16.1 to −1.4)	**0.02**	−4.9±21.9 (−13.1 to 3.3)	0.23	−4.3±21.5 (−12.2 to 3.5)	0.27
ΔSGRQ activity	−2.5±15.0 (−7.7 to 2.7)	0.34	−4.4±17.5 (−11.0 to 2.1)	0.17	−2.6±17.9 (−9.3 to 4.1)	0.43	−2.5±14.8 (−7.9 to 3.0)	0.36
ΔSGRQ impacts	−8.7±16.7 (−14.5 to −2.9)	**<0.01**	−12.9±17.9 (−19.6 to −6.2)	**<0.01**	−10.2±18.4 (−17.1 to −3.4)	**<0.01**	−6.1±20.0 (−13.4 to 1.3)	0.10
ΔLCQ	21.6±32.2 (7.3 to 35.9)	**<0.01**	21.6±29.2 (8.3 to 34.9)	**<0.01**	13.4±24.1 (2.1 to 24.6)	**0.02**	9.1±29.0 (−4.1 to 22.3)	0.17
ΔVAS rest^#^	−3.6±31.5 (−14.8 to 7.5)	0.51	−2.7±25.5 (−12.2 to 6.9)	0.57	−1.1±31.1 (−12.8 to 10.5)	0.85	−0.4±25.4 (−9.7 to 8.9)	0.93
ΔVAS activity^#^	−7.2±22.2 (−15.0 to 0.7)	0.07	−10.3±22.4 (−18.7 to −1.9)	**0.02**	−7.1±25.2 (−17.3 to 1.9)	0.13	−6.7±21.4 (−14.6 to 1.2)	0.09
**Mortality score**								
ΔBODE index	−0.1±1.1 (−0.5 to 0.3)	0.54					0.1±1.4 (−0.4 to 0.6)	0.61
**Inflammatory marker**								
ΔFibrinogen mg·dL^−1^	45.2±84.5 (15.2 to 75.1)	**<0.01**					29.3±65.2 (4.5 to 54.1)	**0.02**

Data are presented as mean±sd (95% CI), unless otherwise stated. Bold type represents statistical significance. Two-tailed t-test used to calculate statistical significance between groups, unless otherwise stated. Δ: change; FEV_1_: forced expiratory volume in 1 s; FVC: forced vital capacity; FIV_1_: forced inspiratory volume in 1 s; VC: vital capacity; *R*aw: airway resistance; 6MWD: 6-min walk distance; mMRC: modified Medical Research Council dyspnoea scale; CAT: COPD Assessment Test; SGRQ: St George's Respiratory Questionnaire; LCQ: Leicester Cough Questionnaire; VAS: visual analogue scale; BODE: body mass index, airflow obstruction, dyspnoea and exercise capacity; IQR: interquartile range. ^#^: pre-treatment 1 data used; ^¶^: Wilcoxon matched-pairs signed rank test used to calculate statistical significance between groups.

### Secondary outcomes

#### Lung function and exercise capacity

Over the 12-month follow-up period, FEV_1_ declined modestly: −96.5 mL (95% CI −169.0 to −23.9; p=0.01). There were no statistically significant changes in airways resistance observed (table 2).

The mean change in 6-min walk distance at 9 months, 24.3 m (95% CI −0.4­ to 49.0 m; p=0.05), was just short of that to achieve the minimal clinically important difference (MCID), 26 m [21], but at 12 months had decreased to 8.5 m (95% CI −19.4 to 36.5 m; p=0.54) (table 2).

#### Patient-reported outcomes

The MCID of −4 points in the total SGRQ score was met at the 12-month follow-up. The total SGRQ was driven by “symptoms” and “impact” domains and endured at 9 months: −6.9 (95% CI −13.0 to −0.9; p=0.03) (figure 2 and table 2).

**FIGURE 3:**
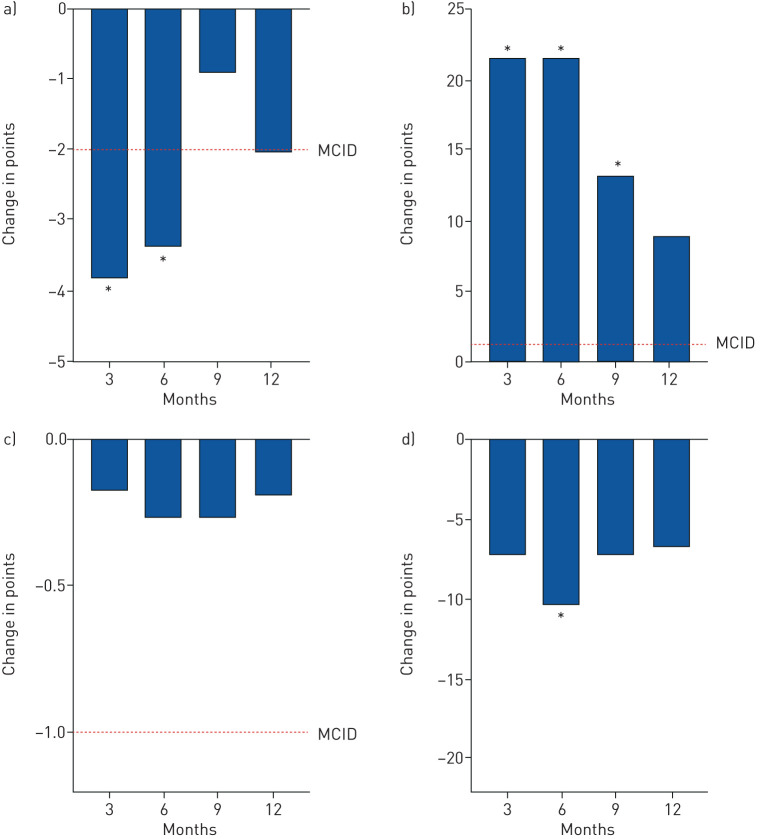
Mean changes in patient-reported outcomes over 12 months. a) COPD Assessment Test score; b) Leicester Cough Questionnaire score; c) modified Medical Research Council dyspnoea score; and d) visual analogue scale (activity). MCID: minimal clinically important difference. *: p<0.05 compared to baseline.

Mean change in CAT was statistically significant and clinically meaningful at 3 and 6 months [22]: −3.8 (95% CI −6.4 to −1.3; p<0.01) and −3.4 (95% CI −5.9 to −0.9; p=0.01), respectively. At 12 months, the MCID of −2 was met, but was not statistically significant: −2.0 (95% CI −4.7 to 0.6; p=0.12) (figure 3 and table 2).

The mean change in LCQ score was statistically significant and far exceeded the MCID of +1.3 [23] at 3, 6 and 9 months: 21.6 (95% CI 7.3 to 35.9; p<0.01), 21.6 (95% CI 8.3 to 34.9; p<0.01) and 13.4 (95% CI 2.1 to 24.6; p=0.02), respectively. At 12 months, the LCQ score exceeded the MCID, but was not statistically significant: 9.1 (95% CI −4.1 to 22.3; p=0.17).

Mean change in VAS on activity was statistically significant at 6 months: −10.3 (95% CI −18.7 to −1.9; p=0.02). There were no statistically significant improvements in mMRC over 12 months.

**FIGURE 4:**
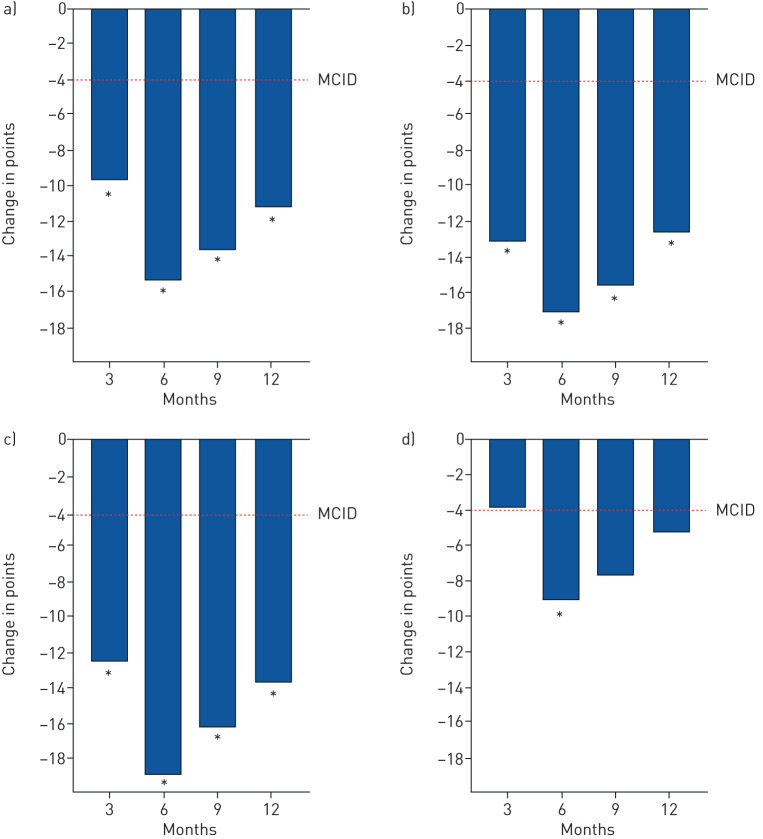
Mean changes in the total St George's Respiratory Questionnaire (SGRQ) total and domain scores over 12 months in those individuals with baseline total SGRQ scores of >50 points. a) SGRQ total score; b) SGRQ symptoms score; c) SGRQ impacts score; d) SGRQ activity score. MCID: minimal clinically important difference. *: p<0.05 compared to baseline.

On *post hoc* analysis, those individuals who had worse baseline SGRQ total scores (*i.e.* >50 points) experienced substantially greater improvements at 3, 6, 9 and 12 months: ΔSGRQ-total scores of −9.8 (95% CI −15.9 to −3.8), −15.4 (95% CI −22.6 to −8.2), −13.5 (95% CI −20.7 to −6.3) and −10.9 (95% CI −16.4 to −5.4), respectively (p<0.01 at all time points) (figure 4 and supplementary table S9), not attributable to regression to the mean on ANCOVA analysis (p=0.29); ΔCAT scores of −5.2 (95% CI −8.4 to −2.1; p<0.01), −5.4 (95% CI −8.6 to −2.3; p<0.01), −2.2 (95% CI −6.2 to 1.8; p=0.27) and −4.0 (95% CI −7.2 to −0.8; p=0.02), respectively; ΔLCQ scores of 36.3 (95% CI 20.1 to 52.5), 35.0 (95% CI 17.4 to 52.6), 26.2 (95% CI 12.7 to 39.6) and 23.5 (95% CI 10.2 to 36.9), respectively (p<0.01 at all time points); and ΔVAS (activity) of −10.6 (95% CI −21.4 to 0.3; p=0.06), −15.8 (95% CI −27.6 to −4.1; p=0.01), −11.9 (95% CI −25.5 to 1.7; p=0.08) and −10.9 (95% CI −22.0 to 0.2; p=0.05), respectively (supplementary table S10).

#### Bronchoscopy outcomes

The presence of mucus at each bronchoscopy was documented as none, mild, moderate and severe, as follows. Treatment 1: 0%, 49%, 37% and 14%, respectively; treatment 2: 9%, 35%, 41% and 15%, respectively; and treatment 3: 0%, 65%, 29% and 6%, respectively.

Microbiology samples obtained for Gram stain (bacteria, mycobacteria and fungi) were evaluated as follows. Treatment 1: 22.9%, 0% and 14.3%, respectively; treatment 2: 26.5%, 2.9% and 23.5%, respectively; and treatment 3: 20.6%, 8.8% and 23.5%, respectively.

128 endobronchial biopsies from 11 subjects were analysed, including 52 at baseline and 57 post-treatment (at day 60). No definitive histological differences were observed.

## Discussion

We have shown that MCS administered to patients with chronic bronchitis in COPD produced statistically significant and clinically meaningful improvements in patient-reported outcomes at 3 months. The reduction in total SGRQ score was driven by “symptoms” and “impact” domains and was durable at 9 months. The symptoms domain includes the assessment of cough and sputum production, which the RejuvenAir system is designed to ameliorate, and has been suggested as a robust descriptor of the chronic bronchitic phenotype prone to exacerbations [24]. The reduction in SGRQ total score was accompanied by clinically relevant gains in CAT and LCQ scores at 6 and 9 months, respectively, reinforcing the beneficial impact of MCS treatment on multidimensional disease-specific and treatment-responsive patient-reported outcomes evaluating cough and sputum production. Subjects with poorer baseline health status (defined as a total SGRQ score of >50 points) experienced substantially greater benefits in these domains that persisted out to 12 months and which may inform future patient selection.

The use of MCS therapy was safe and feasible. All but one subject completed the three treatments and the ratio of full-dose sprays exceeded 84% at each of the procedures. None of the device observations resulted in an adverse event and the majority were resolved by replacing the catheter. All patients were fit for discharge on the day of their treatment. The treatment was safe, with 2.4% of adverse events related to the device and 15.9% to the procedure, all were mild or moderate, and resolved without sequelae. There were no device- or procedure-related serious adverse events. The RejuvenAir system is intended to induce a regenerative endobronchial tissue effect by 1) destroying abnormal surface epithelium with mucin-producing goblet cell hyperplasia; 2) promoting normal ciliated bronchial epithelium regrowth without globlet cell hyperplasia; and 3) reducing chronic inflammation and associated airway constriction. The modest decline in FEV_1_ observed might reflect the epithelial-focused nature of this treatment to airways that have since remodelled on a background of natural disease progression [25].

Most of the safety events were related to natural progression of their disease or unrelated medical disorder. Post-treatment exacerbation frequency increased with GOLD grade, consistent with the experiences of others in the literature [26]. From completion of treatment 1 to 12 months, the exacerbation rates of subjects classified as GOLD grades II and III were 1.29 and 2.10 per patient-year, respectively. These rates compare favourably to those reported in untreated similarly matched individuals: 2.68 per patient-year in GOLD grade II and 3.43 per patient-year in GOLD grade III [27]. Higher baseline total SGRQ score was associated with an increased exacerbation rate and this mirrors a large dataset of 12 043 patients in whom a higher SGRQ total score predicted increased risk of an adverse COPD outcome (exacerbations, hospitalisation or death) [28]. A reduction in SGRQ achieved using the RejuvenAir MCS treatment may translate to a reduction in COPD exacerbations, particularly in more symptomatic individuals [24], although this is speculative.

The study had some limitations. In the interest of risk adversity, there was a prolonged interval of 9.4 (8.7–10.8) months between the first and third treatments in the initial 11 (phase A) patients, which may have influenced the efficacy of the therapy and skewed the overall 12-month outcomes, potentially diluting the effects on patient-reported outcomes demonstrated in this study. Multiple validated, but nevertheless subjective, disease-specific instruments (SGRQ, CAT and LCQ) were necessary to characterise complex symptoms such as cough, sputum production, breathlessness and health-related quality of life and their responses to a therapeutic intervention that could not be achieved using any one physiological correlate [29]. The sample size was small, the treatment was unblinded, and a control group was lacking. Moreover, there were no consistent historical data on pre-treatment exacerbation rates. Lastly, no definitive histological differences were observed between baseline and day 60 endobronchial biopsies and may reflect nonuniform sampling as cryothermic sites were not directly marked or grossly identifiable. The forceps biopsies were obtained from the right lower lobe segmental carina and were of varying quality, with crush artefacts. Furthermore, the samples were obtained from mucosal tissue at the carina where there tend to be fewer goblet cells. A more standardised approach within a sham controlled study and sampling using endobronchial cryobiopsies has been initiated and should provide more informative results (ClinicalTrials.gov NCT03892694).

Bronchial rheoplasty is an alternative novel bronchoscopic therapy using pulsed electric fields to ablate the mucosal lining and is currently under investigation [30]. However, no comparable treatment option exists in the mainstream management of chronic bronchitis and current therapeutic modalities are principally pharmacology based. The effects of RejuvenAir MCS on health-related quality of life may be superior compared to mucolytics [31], prophylactic antibiotics [32], inhaled bronchodilators and steroids [33]. Future studies including a randomised sham-controlled trial are advocated to confirm the benefits and durability of this treatment in a larger population of patients.

### Conclusions

Treatment with the RejuvenAir system in individuals with chronic bronchitis in COPD is safe, feasible, well tolerated, and resulted in clinically and statistically meaningful improvements in multidimensional measures of cough, sputum production, breathlessness and health-related quality of life. The safety and efficacy of this therapy will require confirmation by prospective randomised, sham-controlled trials.

## Supplementary material

10.1183/13993003.00556-2020.Supp1 **Please note:** supplementary material is not edited by the Editorial Office, and is uploaded as it has been supplied by the author.
Supplementary material ERJ-00556-2020.SUPPLEMENT

## Shareable PDF

10.1183/13993003.00556-2020.Shareable1 This one-page PDF can be shared freely online.
Shareable PDF ERJ-00556-2020.Shareable
