# Supplementary material for: A prospective safety and feasibility study of metered cryospray for patients with chronic bronchitis in COPD
Source: Eur Respir J. 2020 Dec 17;56(6):2000556. doi: 10.1183/13993003.00556-2020 (PMC7744607; doi:10.1183/13993003.00556-2020)
Supplement: Supplementary file 1 [file ERJ-00556-2020.SUPPLEMENT.pdf]

ONLINE SUPPLEMENT

Section 1

Table S1 (legend): Inclusion and Exclusion criteria.

**Inclusion Criteria**
- Males and females ≥40 to ≤75 years of age.<br>- Subject is able to read, understand, and sign a written Informed Consent in order to participate in the Study.<br>- Subject has been optimally treated according to Gold treatment guidelines without successful resolution of chronic bronchitis and agrees to continue maintenance pulmonary/COPD medications for the duration of the study.<br>- Diagnosis of chronic bronchitis (CB) and chronic obstructive pulmonary disease (COPD) for a minimum of two years. (Chronic Bronchitis is defined clinically as chronic productive cough for 3 months in each of 2 successive years in a patient in whom other causes of productive cough have been excluded.)<br>- Pre-procedure post bronchodilator FEV1 of greater than or equal to 30% and less than or equal to 80% of predicted within 3 months of enrolment.<br>- Smoking history of at least 10 pack years.<br>- Non-smoking for a minimum of 2 months prior to consent and agrees to continue not smoking for the duration of the study.<br>- Subject is able to adhere to and undergo 3 (4 if in Phase A) bronchoscope procedures that includes lung biopsies and multiple MCS treatments in the opinion of the investigator or per hospital guidelines. (Only Phase A subjects receive biopsies).
**Exclusion Criteria**
- Subject has had an acute pulmonary infection or pneumonia within prior 6 weeks of study bronchoscopy.<br>- Subject has had a CB and/or COPD exacerbation (requiring steroids and/or antibiotics) within 6 weeks prior to study bronchoscopy, as defined by their treating physician.<br>- Subject has clinically significant bronchiectasis or other respiratory disease other than chronic bronchitis and COPD; subject with chronic cough of other pathogenesis, in particular cardiac cause. Subject with the following conditions should not undergo bronchoscopy: untreatable or life-threatening arrhythmias, inability to adequately oxygenate the patient during the procedure, or subject has acute respiratory failure with hypercapnia (unless intubated and ventilated) or has high grade tracheal stenosis.<br>- Diagnosis of asthma with an onset before 30 years of age.<br>- Subject has bullous emphysema characterized as large bullae >30 millimetres on CT; or subject has stenosis in the tracheobronchial system, tracheobronchomegaly, trachea-bronchomalacia, amyloidosis or cystic fibrosis. If a CT is not available in the past 12 months, the Principle Investigator may use the baseline HRCT in lieu of the CT.<br>- Subject has had a transplant.<br>- Subject has the inability to walk >140 meters.<br>- Subject has PaC02 >8kPa, or a PaO2<7kPa at room air.<br>- Subject has a RVSP >45mmHg or a LVEF<45% on 2D-cardiac echo.<br>- Subject has a known mucosal tear, requires treatment to the Right Middle Lobe or has undergone lung surgery: pneumonectomy, lobectomy, bullectomy, lung volume reduction surgery.<br>- Subject has had a prior lung device procedure, including emphysema stent(s) implanted, lung coils, valves, lung denervation or other devices for emphysema.<br>- Subject is unable to temporarily discontinue use of anticoagulant therapy: warfarin, Coumadin, LMWH, heparin, clopidrogel (or equal).<br>- Subject is on >10 mg of prednisolone/day.<br>- Subject has a serious medical condition, such as: uncontrolled coagulopathy or bleeding disorder, congestive heart failure, uncontrolled angina, myocardial infarction in the past year, renal failure, liver disease, cerebrovascular accident within the past 6 months, uncontrolled diabetes uncontrolled hypertension, autoimmune disease or uncontrolled gastric reflux.<br>- Subject is pregnant, nursing, or planning to get pregnant during study duration.<br>- Subject has or is receiving chemotherapy or active radiation therapy within the past 6 months or is expected to receive chemotherapy during participation in this study. Subject life expectancy is less than one year.<br>- Subject is or has been in another clinical investigational study within 6 weeks of baseline.<br>- Subject has known sensitivity to medication required to perform bronchoscopy (such as lidocaine, atropine, and benzodiazepines).
CB, Chronic Bronchitis; COPD, Chronic Obstructive Pulmonary Disease; CT, Computed Tomography; FEV1, Forced Expiratory Volume in 1 second; kPA, kilopascal; LVEF, Left Ventricular Ejection Fraction; LMWH, Low Molecular Weight Heparin; MCS, Metered CryoSpray; mg, milligram; mm Hg, millilitre of mercury; $PaCO_2$, Partial pressure of carbon dioxide; PaO_2, Partial pressure of oxygen; RVSP, Right Ventricular Systolic Pressure; 2D, 2-Dimensional.

Table S2 (legend): Procedural details.

	Treatment 1	Treatment 2	Treatment 3	Mean of Treatments
Mean treatment duration (minutes)	33.3 ± 11.8	31.4 ± 11.5	38.3 ± 12.3	34.3 ± 12.1
Mean number of metered cryosprays (MCS)	17.3 ± 4.6	17.6 ± 2.1	26.2 ± 5.8	20.3 ± 6.0
Mean number of Full Doses (sprays)	15.2 ± 4.5	14.9 ± 2.5	21.8 ± 5.4	17.3 ± 5.3
Mean number of Partial Doses (sprays)	2.2 ± 2.5	2.6 ± 2.4	4.4 ± 4.2	3.1 ± 3.3
Mean percentage of Full Doses (%)	87.7	85.3	84.3	85.8
Mean percentage of Partial Doses (%)	12.3	14.7	15.7	14.2

Table S3 (legend): Subject overview of adverse events over 12-months.

Adverse Event (AE) categorisation	N	%
Subjects experiencing any AE	35	100
Subjects experiencing a Serious AE	11	31.4
Subjects experiencing a Device-related AE*	4	11.4
Subjects experiencing a Serious Device Related AE*	0	0
Subjects experiencing a Procedure Related AE*	21	60.0
Subjects experiencing a Serious Procedure Related AE*	0	0
Subjects experiencing a Severe AE**	6	17.1
Subjects experiencing an AE leading to discontinuation***	1	2.9

* = AE is related if: relation to device / procedure is reported 'Possibly', 'Probably' or 'Causal Relationship'.
** = AE is severe if: severity is reported 'Severe'.
*** = AE led to discontinuation if: reason for early withdrawal at End of Study is 'Adverse Event' or 'Death'.

Table S4 (legend): Categorization of adverse events over 12-months.

**Adverse Event (AE) categorisation**		**N**	**%**
Serious	*No*	237	94.4
	Yes	14	5.6
Causality			
	Concomitant or previous medication	8	3.2
	Disease under study	123	49.0
	Medical history	37	14.7
	Other	83	33.1
Relationship to device*			
	Not related	245	97.6
	Possibly	5	2.0
	Probably	1	0.4
Relationship to study procedure*			
	Not related	205	81.7
	Unlikely	6	2.4
	Possibly	30	12.0
	Probably	10	4.0
Severity**			
	Mild	114	45.4
	Moderate	129	51.4
	Severe	8	3.2
Outcome			
	Death	1	0.4
	Ongoing	21	8.4
	Resolved with sequelae	15	6.0
	Resolved without sequelae	214	85.3

* = AE is related if: relation to device or procedure is reported 'Possibly', 'Probably' or 'Causal Relationship'.
** = AE is severe if: severity is reported 'Severe'

Table S5 (legend): Individual classification of adverse events (AEs) over 12-months.

Adverse Event (AE) - System Organ Classification	Total AEs		Total Subjects	
(listed alphabetically)	N	%	N	%
**Ear and labyrinth disorders**	1	0.4	1	2.9
Hypoacusis	1	0.4	1	2.9
Vertigo				
**Gastrointestinal disorders**				
Abdominal pain upper	1	0.4	1	2.9
Diarrhoea	2	0.8	2	5.7
Gastrointestinal disorder	1	0.4	1	2.9
Glossodynia	1	0.4	1	2.9
Nausea	4	1.6	4	11.4
Peptic ulcer	1	0.4	1	2.9
Rectal haemorrhage	1	0.4	1	2.9
Rectal ulcer	1	0.4	1	2.9
Toothache	1	0.4	1	2.9
**General disorders**				
Chest discomfort	3	1.2	2	5.7
Chest pain	4	1.6	4	11
Fatigue	4	1.6	4	11
Pain	1	0.4	1	2.9
Peripheral swelling	1	0.4	1	2.9
**Hepatobiliary disorders**				
Bile duct obstruction	1	0.4	1	2.9
**Infections and infestations**				
Bacterial infection	1	0.4	1	2.9
Cellulitis	2	0.8	2	5.7
Cystitis	2	0.8	2	5.7
Haemophilus infection	1	0.4	1	2.9
Influenza	3	1.2	3	8.6
Nasopharyngitis	5	2.0	3	8.6
Pneumonia	6	2.4	6	17
Pseudomonas infection	1	0.4	1	2.9
Respiratory tract infection	1	0.4	1	2.9
Rhinitis	1	0.4	1	2.9
Sinusitis	2	0.8	2	5.7
Staphylococcal infection	1	0.4	1	2.9
Upper respiratory tract infection	1	0.4	1	2.9
Urinary tract infection	3	1.2	2	5.7
Urosepsis	1	0.4	1	2.9
**Injury, poisoning and procedural complications**				
Fall	1	0.4	1	2.9
Procedural hypotension	1	0.4	1	2.9
Rib fracture	1	0.4	1	2.9
Skin abrasion	1	0.4	1	2.9
Wound complication	1	0.4	1	2.9
**Investigations**				
Bacterial test positive	1	0.4	1	2.9
Blood potassium decreased	1	0.4	1	2.9
Blood sodium decreased	1	0.4	1	2.9
Moraxella test positive	1	0.4	1	2.9
Mycobacterium test positive	2	0.8	2	5.7

Adverse Event (AE) - System Organ Classification	Total AEs		Total Subjects	
(listed alphabetically)	N	%	N	%
Pseudomonas test positive	1	0.4	1	2.9
Sputum culture positive	1	0.4	1	2.9
Streptococcus test positive	1	0.4	1	2.9
Vitamin D decreased	1	0.4	1	2.9
**Metabolism and nutrition disorders**				
Hypoglycaemia	2	0.8	1	2.9
**Musculoskeletal and connective tissue disorders**				
Arthralgia	2	0.8	2	5.7
Back pain	1	0.4	1	2.9
Bursitis	1	0.4	1	2.9
Dupuytren’s contracture	1	0.4	1	2.9
Joint swelling	2	0.8	2	5.7
Muscle spasms	1	0.4	1	2.9
Musculoskeletal discomfort	1	0.4	1	2.9
Musculoskeletal pain	3	1.2	3	8.6
Neck pain	1	0.4	1	2.9
Pain in extremity	1	0.4	1	2.9
Polymyalgia rheumatica	1	0.4	1	2.9
**Neoplasms benign, malignant**				
Malignant melanoma	1	0.4	1	2.9
Skin cancer	1	0.4	1	2.9
**Nervous system disorders**				
Balance disorder	1	0.4	1	2.9
Headache	1	0.4	1	2.9
Migraine	1	0.4	1	2.9
Morton’s neuralgia	1	0.4	1	2.9
Nervous system disorder	1	0.4	1	2.9
**Psychiatric disorders**				
Anxiety	1	0.4	1	2.9
**Renal and urinary disorders**				
Chronic kidney disease	1	0.4	1	2.9
Dysuria	1	0.4	1	2.9
Urinary retention	2	0.8	2	5.7
**Reproductive system and breast disorders**				
Breast mass	1	0.4	1	2.9
Pelvis prolapse	1	0.4	1	2.9
**Respiratory, thoracic and mediastinal disorders**				
Bronchospasm	1	0.4	1	2.9
Chronic obstructive pulmonary disease	91	36.3	32	91.4
Cough	5	2.0	4	11.4
Dyspnoea	8	3.2	7	20.0
Epistaxis	2	0.8	2	5.7
Hyperventilation	1	0.4	1	2.9
Increased viscosity of bronchial secretion	1	0.4	1	2.9
Lung consolidation	2	0.8	2	5.7
Oropharyngeal pain	1	0.4	1	2.9
Pulmonary embolism	1	0.4	1	2.9
Pulmonary mass	8	3.2	8	22.9
Rhinorrhoea	4	1.6	4	11.4
Sputum increased	3	1.2	3	8.6
Wheezing	4	1.6	2	5.7

Adverse Event (AE) - System Organ Classification	Total AEs		Total Subjects	
(listed alphabetically)	N	%	N	%
**Skin and subcutaneous tissue disorders**				
Eczema	1	0.4	1	2.9
Erythema	1	0.4	1	2.9
**Surgical and medical procedures**				
Cholecystectomy	1	0.4	1	2.9
Hip arthroplasty	1	0.4	1	2.9
Knee operation	1	0.4	1	2.9
Tooth extraction	1	0.4	1	2.9
**Vascular disorders**				
Aortic aneurysm	2	0.8	2	5.7
Hypertension	1	0.4	1	2.9
Hypotension	3	1.2	3	8.6
Thrombophlebitis	1	0.4	1	2.9
**Total**	**251**	**100**	**35**	**100**

Table S6 (legend): Device-related events.

Subject	Adverse Event	Severity	Duration (days)	Outcome	Device	Procedure
47-011	Exacerbation of COPD	Mild	7	Resolved without sequelae	Possibly	Possibly
47-011	Bronchospasm	Moderate	0	Resolved without sequelae	Probable	Probable
47-012	Exacerbation of COPD	Moderate	15	Resolved without sequelae	Possibly	Possibly
47-014	Exacerbation of COPD	Moderate	37	Resolved without sequelae	Possibly	Possibly
47-014	Exacerbation of COPD	Moderate	25	Resolved without sequelae	Possibly	Possibly
47-034	Exacerbation of COPD	Mild	14	Resolved without sequelae	Possibly	Possibly

Table S7 (legend): Serious adverse events over 12-months.

Subject ID	Description of SAE	Duration (days)	Outcome	Severity	Related to device	Related to procedure
46-003	Cough increased	72	Resolved without sequelae	Moderate	Not Related	Not Related
46-004	COPD exacerbation	9	Resolved without sequelae	Moderate	Not Related	Not Related
46-005	COPD exacerbation	6	Resolved without sequelae	Moderate	Not Related	Not Related
46-006	COPD exacerbation	39	Resolved without sequelae	Moderate	Not Related	Not Related
47-002	Peptic ulcer	117	Resolved without sequelae	Severe	Not Related	Not Related
47-008	Pulmonary embolus	4	Resolved with sequelae	Severe	Not Related	Not Related
47-008	Chest pain	39	Death	Severe	Not Related	Not Related
47-008	Rectal bleeding	1	Resolved without sequelae	Severe	Not Related	Not Related
47-014	COPD exacerbation	10	Resolved without sequelae	Severe	Not Related	Not Related
47-017	Pneumonia	12	Resolved without sequelae	Severe	Not Related	Not Related
47-034	COPD exacerbation	29	Resolved without sequelae	Severe	Not Related	Not Related
51-003	Pneumonia	170	Resolved without sequelae	Severe	Not Related	Not Related
51-006	COPD exacerbation	4	Resolved without sequelae	Moderate	Not Related	Not Related
51-006	Urosepsis	19	Resolved without sequelae	Moderate	Not Related	Not Related

SAE = Serious Adverse Event

Table S8 (legend): Exacerbation rates over 12-months.

Time period	N	Per patient year
Whole cohort		
T1 to 3-months	49	2.00
T1 to 6-months	59	2.04
T1 to 9-months	65	1.81
T1 to 12-months	83	1.84
T3 to 3-months	22	2.50
T3 to 6-months	34	2.09
T3 to 9-months	45	1.98
T3 to 12-months	62	1.96
GOLD grade II		
T1 to 3-months	17	1.89
T1 to 6-months	18	1.72
T1 to 9-months	19	1.46
T1 to 12-months	21	1.29
T3 to 3-months	7	2.32
T3 to 6-months	9	1.50
T3 to 9-months	12	1.43
T3 to 12-months	14	1.19
GOLD grade III		
T1 to 3-months	32	2.03
T1 to 6-months	41	2.29
T1 to 9-months	46	2.01
T1 to 12-months	62	2.10
T3 to 3-months	15	2.44
T3 to 6-months	25	2.41
T3 to 9-months	33	2.24
T3 to 12-months	48	2.28

GOLD, Global initiative for Obstructive Lung Disease; T1, Treatment 1; T3, Treatment 3.

Table S9 (legend): Changes in St George's Respiratory Questionnaire total scores over 12-months stratified according to disease severity thresholds.

		3-month		6-month		9-month		12-month	
		value	*p-value*	value	*p-value*	value	*p-value*	*value*	*p-value*
Baseline SGRQ-Total Score > 50 points		n=24		n=20		n=20		n=21	
∆SGRQ	*Total score*	-9.8 ± 14.4	**<0.01**	-15.4 ± 15.3	**<0.01**	-13.5 ± 15.4	**<0.01**	-10.9 ± 12.1	**<0.01**
		(95% CI -15.9 to -3.8)		(95% CI -22.6 to -8.2)		(95% CI -20.7 to -6.3)		(95% CI -16.4 to -5.4)	
	Symptoms	-13.2 ± 19.0	**<0.01**	-17.2 ± 16.7	**<0.01**	-15.6 ± 16.9	**<0.01**	-12.8 ± 17.9	**<0.01**
		(95% CI -21.2 to -5.2)		(95% CI -25.0 to -9.4)		(95% CI -23.5 to -7.7)		(95% CI -20.9 to -4.6)	
	Activity	-3.9 ± 15.0	0.21	-9.0 ± 17.2	**0.03**	-7.8 ± 18.3	0.07	-5.3 ± 16.1	0.15
		(95% CI -10.2 to 2.4)		(95% CI -17.1 to -1.0)		(95% CI -16.4 to 0.7)		(95% CI -12.7 to 2.0)	
	Impacts	-12.5 ± 17.7	**<0.01**	-18.9 ± 18.7	**<0.01**	-16.4 ± 19.3	**<0.01**	-13.8 ± 16.9	**<0.01**
		(95% CI -19.9 to -5.0)		(95% CI -27.6 to -10.1)		(95% CI -25.4 to -7.3)		(95% CI -21.5 to -6.1)	
Baseline SGRQ-Total Score < 50 points		n=10		n=10		n=10		n=10	
∆SGRQ	*Total score*	2.0 ± 10.9	0.58	2.3 ± 8.4	0.41	6.3 ± 7.2	**0.02**	8.6 ± 12.1	0.05

		(95% CI -5.8 to 9.8)		(95% CI -3.7 to 8.3)		(95% CI 1.1 to 11.4)		(95% CI 0.0 to 17.3)	
	Symptoms	10.2 ± 20.8	0.15	8.2 ± 13.3	0.09	16.6 ± 13.4	**<0.01**	13.4 ± 17.6	**0.04**
		(95% CI -4.6 to 25.0)		(95% CI -1.4, 17.7)		(95% CI 7.0 to 26.2)		(95% CI 0.8 to 26.0)	
	Activity	0.9 ± 15.3	0.86	4.8 ± 14.8	0.33	7.8 ± 12.1	0.07	3.5 ± 9.9	0.29
		(95% CI -10.0 to 11.8)		(95% CI -5.8 to 15.4)		(95% CI -0.8 to 16.4)		(95% CI -3.5 to 10.6)	
	Impacts	0.3 ± 9.9	0.92	-1.0 ± 7.3	0.68	2.0 ± 7.1	0.39	10.2 ± 16.3	0.08
		(95% CI -6.8 to 7.4)		(95% CI -6.2 to 4.3)		(95% CI -3.1 to 7.1)		(95% CI -1.5 to 21.8)	
<u>Baseline CAT Score > 10 points</u>		n=31		n=28		n=28		n=29	
△SGRQ	*Total score*	-6.2 ± 14.9	**0.03**	-10.0 ± 16.2	**<0.01**	-7.6 ± 16.5	**0.02**	-5.5 ± 15.1	0.06
		(95% CI -11.7 to -0.7)		(95% CI -16.3 to -3.7)		(95% CI -14.0 to -1.3)		(95% CI -11.2 to 0.3)	
	Symptoms	-5.7 ± 22.2	0.16	-8.7 ± 20.3	**0.03**	-5.7 ± 22.2	0.19	-5.8 ± 21.3	0.16
		(95% CI -13.8 to 2.4)		(95% CI -16.6 to -0.8)		(95% CI -14.3 to 3.0)		(95% CI -13.9 to 2.3)	
	Activity	-2.4 ± 15.4	0.39	-4.8 ± 18.0	0.17	-3.1 ± 18.4	0.39	-2.0 ± 15.2	0.48
		(95% CI -8.0 to 3.2)		(95% CI -11.7 to 2.2)		(95% CI -10.2 to 4.1)		(95% CI -7.8 to 3.8)	
	Impacts	-8.7 ± 17.4	**0.01**	-13.6 ± 18.3	**<0.01**	-11.0 ± 18.8	**<0.01**	-7.4 ± 19.5	0.05
		(95% CI -15.1 to -2.3)		(95% CI -20.7 to -		(95% CI -18.3 to -3.7)		(95% CI -14.9 to	

				6.5)				0.0)	
Baseline CAT Score < 10 points		n=2		n=2		n=2		n=2	
△SGRQ	*Total score*	-4.8 ± 6.9 (95% CI -66.8 to 57.1)	0.50	-2.3 ± 3.5 (95% CI -33.6 to 29.1)	0.53	3.0 ± 5.2 (95% CI -43.7 to 49.7)	0.56	7.3 ± 11.4 (95% CI -95.1 to 109.7)	0.53
	Symptoms	0.7 ± 10.3 (95% CI -91.5 to 92.9)	0.94	-9.7 ± 5.2 (95% CI -56.8 to 37.4)	0.23	6.2 ± 18.2 (95% CI -157.6 to 169.9)	0.72	16.5 ± 13.7 (95% CI -107.0 to 140.0)	0.34
	Activity	-8.9 ± 12.9 (95% CI -125.2 to 107.4)	0.51	0.4 ± 9.1 (95% CI -81.6 to 82.4)	0.96	3.5 ± 4.7 (95% CI -38.4 to 45.5)	0.48	-8.7 ± 4.0 (95% CI -44.4 to 26.9)	0.20
	Impacts	-3.9 ± 2.5 (95% CI -26.0 to 18.1)	0.27	-2.6 ± 0.5 (95% CI -7.3 to 2.1)	0.09	0.3 ± 2.0 (95% CI -18.0 to 18.6)	0.86	13.9 ± 21.2 (95% CI -176.2 to 203.9)	0.52
Baseline GOLD grade of 3		n=22		n=19		n=19		n=20	
△SGRQ	*Total score*	-6.9 ± 14.4 (95% CI -13.3 to -0.5)	**0.04**	-10.7 ± 15.5 (95% CI -18.2 to -3.3)	**<0.01**	-8.6 ± 16.6 (95% CI -16.6 to -0.6)	**0.04**	-6.3 ± 13.1 (95% CI -12.4 to -0.1)	0.05
	Symptoms	-9.4 ± 23.4 (95% CI -19.8 to 0.9)	0.07	-9.2 ± 20.2 (95% CI -18.9 to 0.6)	0.06	-7.2 ± 23.0 (95% CI -18.3 to 3.9)	0.19	-7.1 ± 20.1 (95% CI -16.5 to 2.3)	0.13

	Activity	-1.5 ± 14.3	0.63	-4.7 ± 16.2	0.22	-2.8 ± 16.1	0.46	-1.0 ± 10.2	0.66
		(95% CI -7.9 to 4.9)		(95% CI -12.5 to 3.1)		(95% CI -10.5 to 5.0)		(95% CI -5.8 to 3.8)	
	Impacts	-9.2 ± 16.2	**0.01**	-14.7 ± 17.6	**<0.01**	-12.4 ± 20.2	**0.02**	-9.0 ± 18.0	**0.04**
		(95% CI -16.4 to -2.1)		(95% CI -23.2 to -6.3)		(95% CI -22.1 to -2.7)		(95% CI -17.4 to -0.5)	
<u>Baseline GOLD grade of 2</u>		n=10		n=9		n=9		n=9	
△SGRQ	*Total score*	-5.1 ± 16.4	0.35	-8.0 ± 18.5	0.23	-5.7 ± 17.0	0.35	-2.8 ± 19.6	0.68
		(95% CI -16.8 to 6.7)		(95% CI -22.2 to 6.2)		(95% CI -18.7 to 7.4)		(95% CI -17.9 to 12.2)	
	Symptoms	0.42 ± 20.9	0.95	-8.5 ± 21.8	0.28	-4.1 ± 21.0	0.57	-2.3 ± 25.0	0.79
		(95% CI -14.5 to 15.3)		(95% CI -25.2 to 8.3)		(95% CI -20.3 to 12.0)		(95% CI -21.5 to 17.0)	
	Activity	-3.5 ± 17.7	0.54	-5.7 ± 22.3	0.46	-3.8 ± 23.7	0.64	-4.4 ± 23.6	0.59
		(95% CI -16.2 to 9.1)		(95% CI -22.8 to 11.4)		(95% CI -22.0 to 14.4)		(95% CI -22.5 to 13.8)	
	Impacts	-8.0 ± 20.4	0.25	-9.7 ± 20.6	0.19	-7.7 ± 16.5	0.20	-2.6 ± 23.1	0.74
		(95% CI -22.5 to 6.5)		(95% CI -25.5 to 6.1)		(95% CI -20.4 to 5.0)		(95% CI -20.4 to 15.1)	

Numeric data are presented as mean ± SD or median (IQR). Two-tailed t-test or Wilcoxon matched-pairs signed rank test[†] were used, respectively, to calculate statistical significance between groups. [§]Pre-treatment 1 data used. BMI, Body Mass Index; BODE index = Body mass index, airflow Obstruction, Dyspnoea, and Exercise capacity; CAT, COPD Assessment Test; FEV_1, Forced Expiratory Volume in 1 second; FIV1, Forced Inspiratory Volume in 1 second; FVC, Forced Vital Capacity; GOLD, Global initiative for Obstructive Lung Disease; IC, Inspiratory Capacity; LCQ, Leicester Cough Questionnaire; mMRC, modified Medical Research Council dyspnoea scale; PO_2, Partial pressure for oxygen; RAW, airways resistance; RV, Residual Volume; SGRQ, St George's Respiratory Questionnaire; TLC, total lung capacity; TL_{CO}, transfer factor for carbon monoxide;

VAS, Visual Analogue Score; VC, Vital Capacity; 6MWD, six-minute walk distance.

Figure S5 (legend): Changes in St George's Respiratory Questionnaire total scores over 12-months stratified according to disease severity thresholds: A) SGRQ-Total Score > 50; B) SGRQ-Total Score < 50; C) CAT score > 10; D) CAT score < 10; E) Baseline GOLD grade 3; F) Baseline GOLD grade 2.

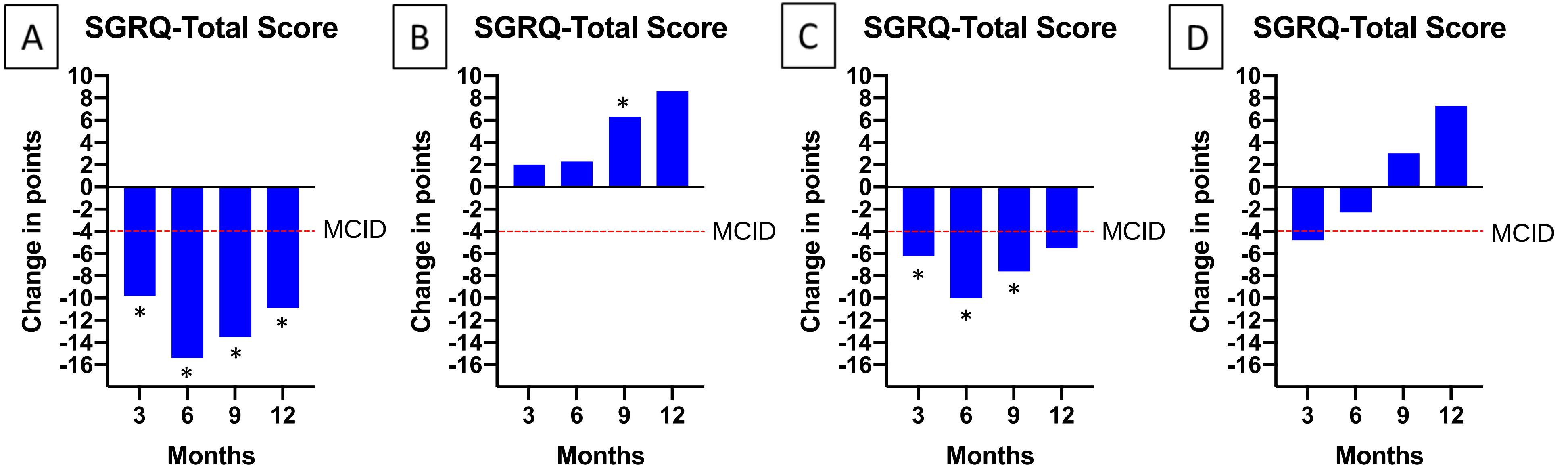

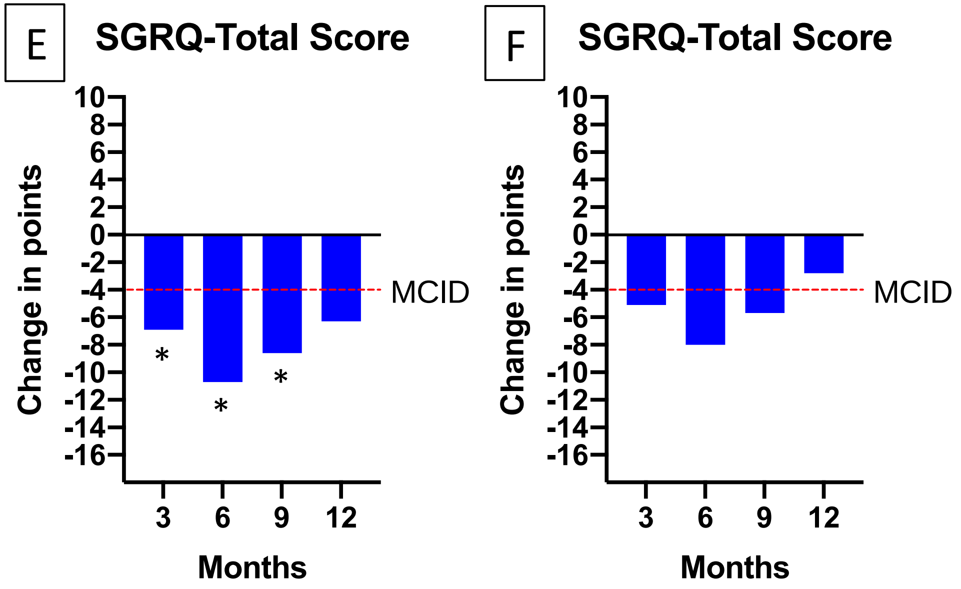
E
SGRQ-Total Score
Change in points
10
8
6
4
2
0
-2
-4
-6
-8
-10
-12
-14
-16
MCID
*
*
*
3
6
9
12
Months
F
SGRQ-Total Score
Change in points
10
8
6
4
2
0
-2
-4
-6
-8
-10
-12
-14
-16
MCID
3
6
9
12
Months

Table S10 (legend): Changes in Patient-Reported Outcomes over 12-months in those individuals with a baseline SGRQ total score > 50 points.

Baseline SGRQ-Total Score > 50 points	3-month value	*p-value*	6-month value	*p-value*	9-month value	*p-value*	12-month *value*	*p-value*
△CAT[§]	n=23<br>-5.2 ± 7.3<br>(95% CI -8.4 to -2.1)	**<0.01**	n=20<br>-5.4 ± 6.8<br>(95% CI -8.6 to -2.3)	**<0.01**	n=20<br>-2.2 ± 8.6<br>(95% CI -6.2 to 1.8)	0.27	n=21<br>-4.0 ± 7.0<br>(95% CI -7.2 to -0.8)	**0.02**
△LCQ	n=14<br>36.3 ± 28.1<br>(95% CI 20.1 to 52.5)	**<0.01**	n=13<br>35.0 ± 29.1<br>(95% CI 17.4 to 52.6)	**<0.01**	n=12<br>26.2 ± 21.1<br>(95% CI 12.7 to 39.6)	**<0.01**	n=13<br>23.5 ± 22.1<br>(95% CI 10.2 to 36.9)	**<0.01**
△mMRC	n=24<br>0 (-1, 0)<br>(95.7% CI 0 to 0)	0.24	n=20<br>0 (-1, 0)<br>(95.9% CI -1 to 0)	**0.04**	n=20<br>0 (-1, 0)<br>(95.9% CI -1 to 0)	0.13	n=21<br>0 (-1, 0)<br>(97.3% CI -1 to 0)	0.49
△VAS - Rest[§]	n=23<br>-10.0 ± 33.5<br>(95% CI -24.4 to 4.5)	0.17	n=20<br>-7.4 ± 29.9<br>(95% CI -21.4 to 6.6)	0.28	n=20<br>-4.2 ± 36.2<br>(95% CI -21.1 to 12.8)	0.61	n=21<br>-5.8 ± 28.3<br>(95% CI -18.6 to 7.1)	0.36
	n=23		n=20		n=20		n=21	

△VAS - Activity[§]	-10.6 ± 25.1 (95% CI -21.4 to 0.3)	0.06	-15.8 ± 25.0 (95% CI -27.6 to -4.1)	**0.01**	-11.9 ± 29.1 (95% CI -25.5 to 1.7)	0.08	-10.9 ± 24.3 (95% CI -22.0 to 0.2)	0.05

Numeric data are presented as mean ± SD or median (IQR). Two-tailed t-test or Wilcoxon matched-pairs signed rank test[†] were used, respectively, to calculate statistical significance between groups. [§]Pre-treatment 1 data used. CAT, COPD Assessment Test; LCQ, Leicester Cough Questionnaire; mMRC, modified Medical Research Council dyspnoea scale; VAS, Visual Analogue Score.

Section 2

2.1. Methodology - Phases of Study

Phase A

Between 8th March 2016 and the 30th August 2016, eleven subjects were allocated to receive a single treatment to the right lower lobe and main stem bronchus. Six endobronchial biopsies were collected from the first segmental and lobular bronchi immediately prior to MCS delivery. Bronchoscopy was performed again at 60+/-7 days, the biopsy sites identified, guided by photographic documentation, inspected, and the sampling regime repeated. Biopsies were evaluated for evidence of healing and healthy mucosal regeneration. The patients were assessed at the 3-month follow-up visit and the primary endpoint data submitted to the data safety monitoring board (DSMB). Subjects received a monthly telephone call to ascertain their wellbeing and health status until review and approval of Phase A data by the DSMB.

Phase B

Following receipt of a satisfactory report on the findings in Phase A by the DSMB on the 29th September 2016, the participants' schedules were completed with two further sessions, treatments to the left lower lobe and main stem bronchus, followed by treatments to both the upper lobes, any residual main stem bronchus and the distal end of the trachea. (The right middle lobe was not treated). Airways have been inspected at each procedure and video recordings made. Intervals of 30 to 45 days were imposed between sessions and progression to the next treatment was contingent on the subject remaining stable without evidence of a recent acute exacerbation. An additional twenty-four subjects were enrolled and have undergone their three scheduled treatments: the last subject entered on the 6th November 2017. 12-month follow-up was completed on the 14th February 2019.

2.2. Methodology - Patient-Reported Outcome (PRO) Assessment Tools

The St George's Respiratory Questionnaire (SGRQ) is a 50-item multidimensional instrument to measure quality of life in patients with airways obstruction and to quantify changes after therapy(1, 2). Scores are calculated for three domains: Symptoms (frequency and severity), Activities (that cause or are limited by breathlessness), and Impacts (psycho-social disturbance resulting from airways disease), that are combined to generate a total score. Scores range from 0 to 100, with higher scores indicating more severe limitation. An MCID of ≥4 is considered meaningful(3).

The COPD Assessment Test (CAT) is an 8-item multidimensional tool that evaluates the impact of the disease (cough, sputum, dyspnoea, chest tightness) on quality of life(4). CAT scores range from 0 to 40, with higher scores denoting more severe impact on an individual's life. An MCID of ≥2(5) and a triangulated MCID of ≥2.54(6) have been suggested.

The LCQ is a 19-item multidimensional instrument using a 7-point Likert response scale designed to assess the impact of cough on three domains: physical, psychological and social(7). Patients are asked to complete the questionnaire daily for two weeks prior to each follow-up visit. An MCID of ≥1.3 is considered meaningful(8).

The visual analogue scale (VAS) is a unidimensional psychometric measure for subjective characteristics or attitudes that cannot be objectively quantified. The patient's assessment of his or her current state for a given parameter is indicated with a mark on a linear scale representing the worst to the best outcomes. An MCID is not yet established.

The modified Medical Research Council (mMRC) dyspnoea scale(9) provides a simple means of categorising patients in terms of the disability associated with breathlessness due to COPD(10). A minimum clinically important difference (MCID) of ≥1 is considered meaningful(11).

2.3. Methodology - Device

The RejuvenAir® System is a cryosurgical device that delivers metered doses of medical grade liquid nitrogen from a dewar in a console to a catheter emitting a radial spray at its tip(12). A thermocouple at the distal end of the catheter tailors the dosage of spray to the diameter of the targeted airway. The single-use 5.3-French cryo-catheter, with an introducer, is inserted through the 2mm working channel of a standard therapeutic flexible video-bronchoscope with 4.4mm outer diameter (OD) to reach the targeted site and deliver the vaporised LN_2 at about -195°C(13) with a cooling energy of 25W(14). The spray location guide sheath fits over the shaft of the bronchoscope. 0.5cm graduations facilitate accurate deliveries of MCS incrementally throughout the airway tree.

2.4. Methodology – Procedure

The RejuvenAir® procedure is carried out in an operating room or bronchoscopy suite. Enrolled subjects received standard anaesthetic, sedative and associated medications per institutional guidelines and routine clinical practice for their bronchoscopy procedure.

As part of the pre-anaesthetic, glycopyrrolate could be administered to reduce airway secretions. The patient under general anaesthesia is intubated and ventilated with 100% oxygen. The airways are first suctioned clear of mucus using a separate large diameter flexible bronchoscope and the target lobe sampled for routine microbiology using a bronchial wash pre-treatment.

The 4.4mm OD bronchoscope with inserted cryo-catheter is introduced into the endotracheal tube and navigated to the target site as instructed on the console display touchscreen, the catheter extruded several centimetres and the MCS delivered. The catheter is then retracted incrementally by 1cm and at each station a further MCS released.

Treatment 1 delivered MCS to the right lower lobe and main stem bronchus, treatment 2 to the left lower lobe and main stem bronchus, and treatment 3 to both upper lobes, any residual main stem bronchus, and the distal end of the trachea. (The right middle lobe was not treated). Precautionary measures are employed to avoid barotrauma and asphyxia: The spray is emitted at a pressure of less than 1 psi but the expansion of vaporising LN_2 is 696-fold(13, 15). Before each spray the cuff of the endotracheal tube is deflated, and the ventilator disconnected briefly. One-hour post-procedure a chest x-ray is obtained to exclude barotrauma.

Intervals of 30 to 45 days were imposed between sessions and progression to the next treatment was contingent on the subject remaining stable without evidence of a recent acute exacerbation.

2.5. Methodology – Assessment of severity of adverse events (AEs):

a) Mild: Observations and symptoms requiring no intervention.
b) Moderate: Events leading to minimal non-invasive measures.
c) Severe:
    i. Not immediately life-threatening but necessitating hospitalisation
    ii. Life-threatening indicating urgent intervention
    iii. Fatal

ABBREVIATIONS

AE – Adverse Event

°C – Celsius

CAT – COPD Assessment Test

COPD – Chronic Obstructive Pulmonary Disease

DSMB – Data Safety Monitoring Board

LCQ – Leicester Cough Questionnaire

LN_2 – Liquid Nitrogen

MCS = Metered CryoSpray

MCID – Minimal clinically important difference

OD – Outer Diameter

SGRQ – St George's Respiratory Questionnaire

VAS – Visual Analogue Score

W – Watt

REFERENCES

1. Jones PW, Quirk FH, Baveystock CM. The St George's Respiratory Questionnaire. Respiratory medicine. 1991;85 Suppl B:25-31; discussion 3-7.
2. Jones PW, Quirk FH, Baveystock CM, Littlejohns P. A self-complete measure of health status for chronic airflow limitation. The St. George's Respiratory Questionnaire. The American review of respiratory disease. 1992;145(6):1321-7.
3. Jones PW. St. George's Respiratory Questionnaire: MCID. Copd. 2005;2(1):75-9.
4. Jones PW, Harding G, Berry P, Wiklund I, Chen WH, Kline Leidy N. Development and first validation of the COPD Assessment Test. The European respiratory journal. 2009;34(3):648-54.
5. Kon SS, Canavan JL, Jones SE, Nolan CM, Clark AL, Dickson MJ, et al. Minimum clinically important difference for the COPD Assessment Test: a prospective analysis. The Lancet Respiratory medicine. 2014;2(3):195-203.
6. Alma H, de Jong C, Tsiligianni I, Sanderman R, Kocks J, van der Molen T. Clinically relevant differences in COPD health status: systematic review and triangulation. European Respiratory Journal. 2018;52(3):1800412.
7. Birring SS, Prudon B, Carr AJ, Singh SJ, Morgan MDL, Pavord ID. Development of a symptom specific health status measure for patients with chronic cough: Leicester Cough Questionnaire (LCQ). Thorax. 2003;58(4):339-43.
8. Raj AA, Pavord DI, Birring SS. Clinical cough IV:what is the minimal important difference for the Leicester Cough Questionnaire? Handb Exp Pharmacol. 2009(187):311-20.
9. Mahler DA, Wells CK. Evaluation of clinical methods for rating dyspnea. Chest. 1988;93(3):580-6.
10. Bestall JC, Paul EA, Garrod R, Garnham R, Jones PW, Wedzicha JA. Usefulness of the Medical Research Council (MRC) dyspnoea scale as a measure of disability in patients with chronic obstructive pulmonary disease. Thorax. 1999;54(7):581-6.
11. Araújo Oliveira AL, Andrade L, Marques A. Minimal clinically important difference and predictive validity of the mMRC and mBorg in acute exacerbations of COPD. European Respiratory Journal. 2017;50(suppl 61):PA4705.
12. Mulcahey TI, Coad JE, Fan WL, Grasso DJ, Hanley BM, Hawkes HV, et al. Metered Cryospray: a novel uniform, controlled, and consistent in vivo application of liquid nitrogen cryogenic spray. Medical devices (Auckland, NZ). 2017;10:29-41.
13. Lemmon EW, McLinden, M. O., Friend, D. G., & National Institute of Standards and Technology (U.S.). Thermophysical properties of fluid systems. (NIST chemistry webbook.). 1998.
14. Au JT, Carson J, Monette S, Finley DJ. Spray cryotherapy is effective for bronchoscopic, endoscopic and open ablation of thoracic tissues. Interactive cardiovascular and thoracic surgery. 2012;15(4):580-4.
15. Unit Conversion Data for Nitrogen Universal Industrial Gases, Inc. 2019.
