# Supplementary material for: A prospective safety and feasibility study of metered cryospray for patients with chronic bronchitis in COPD
Source: Eur Respir J. 2020 Dec 17;56(6):2000556. doi: 10.1183/13993003.00556-2020 (PMC7744607; doi:10.1183/13993003.00556-2020)
Supplement: Supplementary file 2 [file ERJ-00556-2020.Shareable.pdf]

ORIGINAL ARTICLE
COPD

SHAREABLE PDF

A prospective safety and feasibility study of metered cryospray for patients with chronic bronchitis in COPD

Justin L. Garner[1,2,3], Tawimas Shaipanich[4], Jorine E. Hartman[5], Christopher M. Orton[1,2,3], Cielito Caneja[1,3], Karin Klooster[5], John Thornton[3], Don D. Sin[4], Dirk-Jan Slebos[5] and Pallav L. Shah[1,2,3]

Affiliations: [1]Department of Respiratory Medicine, Royal Brompton Hospital, London, UK. [2]Airways Diseases Section, National Heart and Lung Institute, Imperial College, London, UK. [3]Chelsea and Westminster Hospital, London, UK. [4]St Paul's Hospital, Vancouver, BC, Canada. [5]Department of Pulmonary Diseases, University of Groningen, University Medical Center Groningen, Groningen, The Netherlands.

Correspondence: Pallav L. Shah, Royal Brompton Hospital, Sydney Street, London SW3 6NP, UK. E-mail: pallav.shah@imperial.ac.uk

@ERSpublications
RejuvenAir system treatment for individuals with chronic bronchitis in COPD is safe, feasible, well tolerated, and resulted in clinically meaningful improvements in multidimensional measures of cough, sputum production, breathlessness and quality of life https://bit.ly/30KBfPs

Cite this article as: Garner JL, Shaipanich T, Hartman JE, *et al.* A prospective safety and feasibility study of metered cryospray for patients with chronic bronchitis in COPD. *Eur Respir J* 2020; 56: 2000556 [https://doi.org/10.1183/13993003.00556-2020].

This single-page version can be shared freely online.

ABSTRACT

Background: No currently approved intervention counteracts airway metaplasia and mucus hypersecretion of chronic bronchitis in COPD. However, metered cryospray (MCS) delivering liquid nitrogen to the tracheobronchial airways ablates abnormal epithelium and facilitates healthy mucosal regeneration. The objective of this study was to evaluate the feasibility, efficacy and safety of MCS in chronic bronchitis.

Methods: Patients with a forced expiratory volume in 1 s of 30–80% predicted who were taking optimal medication were recruited. Primary outcomes were feasibility (completion of treatments), efficacy (3-month change in St George's Respiratory Questionnaire (SGRQ)) and safety (incidence of adverse events). Secondary outcomes were lung function, exercise capacity and additional patient-reported outcomes.

Results: 35 patients, 19 male/16 female, aged 47–76 years, Global Initiative for Chronic Obstructive Lung Disease grade I (n=3), II (n=10) and III (n=22), underwent staggered liquid nitrogen treatments to the tracheobronchial tree. 34 patients completed three treatments, each lasting 34.3±12.1 min, separated by 4–6 weeks; one withdrew after the first treatment. ∼1800 doses of MCS were delivered. Clinically meaningful improvements in patient-reported outcomes were observed at 3 months: change in SGRQ −6.4 (95% CI −11.4 to −1.3; p=0.01), COPD Assessment Test (CAT) −3.8 (95% CI −6.4 to −1.3; p<0.01) and Leicester Cough Questionnaire (LCQ) 21.6 (95% CI 7.3 to 35.9; p<0.01). Changes in CAT were durable to 6 months (−3.4, 95% CI −5.9 to −0.9; p=0.01); changes in SGRQ and LCQ were durable to 9 months (−6.9, 95% CI −13.0 to −0.9; p=0.03 and 13.4, 95% CI 2.1 to 24.6; p=0.02, respectively. At 12 months, 14 serious adverse events were recorded in 11 (31.4%) subjects; six (43%) moderate and eight (57%) severe. Nine were respiratory-related: six exacerbations of COPD, two pneumonias and one case of increased coughing; all recovered without sequelae. None were serious device- or procedure-related adverse events.

Conclusion: MCS is safe, feasible and associated with clinically meaningful improvements in multidimensional patient-reported outcomes.

Copyright ©ERS 2020. This version is distributed under the terms of the Creative Commons Attribution Licence 4.0.
